# Discovery and Identification of Serum Succinyl‐Proteome for Postmenopausal Women with Osteoporosis and Osteopenia

**DOI:** 10.1111/os.12519

**Published:** 2019-10-29

**Authors:** Li‐li Zhang, Chun‐wen Li, Kang Liu, Zhong Liu, Bo‐cheng Liang, Yi‐ran Yang, Xiao‐lin Shi

**Affiliations:** ^1^ Department of Pathology The Second Affiliated Hospital Zhejiang University School of Medicine Hangzhou China; ^2^ Department of Diagnostics of Traditional Chinese Medicine, College of Basic Medical Science Zhejiang Chinese Medical University Hangzhou China; ^3^ Department of Osteology The Second Affiliated Hospital of Zhejiang Chinese Medical University Hangzhou China; ^4^ The Second Clinical Medical College Zhejiang Chinese Medical University Hangzhou China

**Keywords:** Succinylation, Post‐Translational Modification, Osteoporosis, Osteopenia

## Abstract

**Objective:**

For the purpose of providing evidence for the treatment of osteoporosis and osteopenia, this study retrospectively identified succinylation‐modified sites and proteins in postmenopausal women, and bioinformatics analysis were performed.

**Methods:**

From January 2016 to June 2018, a total of 30 postmenopausal women aged from 55 to 70 years old were assigned to three groups: 10 cases with osteoporosis; 10 cases with osteopenia; and 10 cases with normal bone mass. Subsequently, the serum samples were collected from all cases for succinyl‐proteome. Measures comprised label‐free quantitative analysis, succinylation enrichment techniques, the liquid chromatograph–mass spectrometer/mass spectrometer (LC‐MS/MS) methods, and bioinformatics.

**Results:**

A total of 113 succinylation sites on 35 proteins were identified based on quantitative information. The variation of the different multiple folds were more than 1.2 times as a significant increase for up‐regulated and less than 1/1.2 times as a significant decrease for down‐regulated. Among the quantified succinylation sites, 66 were up‐regulated and 11 down‐regulated in the Osteopenia/Normal comparison group, 24 were up‐regulated and 44 down‐regulated in the Osteoporosis/Osteopenia comparison group, 45 were up‐regulated and 32 down‐regulated in the Osteoporosis/Normal comparison group. Among the quantified succinylation proteins, 24 were up‐regulated and 7 down‐regulated in the Osteopenia/Normal comparison group, 15 were up‐regulated and 20 down‐regulated in the Osteoporosis/Osteopenia comparison group, 20 were up‐regulated and 17 down‐regulated in the Osteoporosis/Normal comparison group. The percentage of proteins differed in immune response, signaling pathway, proteolysis, lymphocyte, leukocyte, and cell activation. Four differentially expressed proteins (apolipoprotein A‐I, apolipoprotein A‐II, hemoglobin subunit alpha, and haptoglobin) contained quantitative information; they were mediated with receptors, factors, mechanisms, that related to bone metabolism. Hemoglobin subunit alpha was screened for diagnosis of osteopenia.

**Conclusions:**

The succinyl‐proteome experimental data indicated that apolipoprotein A‐I, apolipoprotein A‐II, hemoglobin subunit alpha, and haptoglobin were valuable for diagnosis and treatment in postmenopausal women with osteoporosis and osteopenia.

## Introduction

Osteoporosis, a bone metabolic disease of multiple causation, is characterized by bone loss. Bone mass continues decline after its peak during the postmenopausal period. The process of bone remodeling is another potential concern because bone mineral density(BMD), bone loss and mineral balance changes in older women, especially in those aged 75 and over, who have higher average annual bone loss^1^. Excessive or insufficient bone resorption results in changes in bone micro‐structure, disordered arrangement of bone trabeculae, and decreased mechanical properties of bone^2^. BMD has been used to diagnose osteoporosis, predict the risk of osteoporotic fracture, monitor the natural course of disease and evaluate the efficacy of drugs. Perhaps the most serious limitations of this method lie in that it does not explain why early symptoms of osteoporosis are unremarkable. Patients may know they are at high risk of suffering from osteoporosis after a primary fracture, but unexpectedly, their BMD values not conform to the standard of osteopenia or osteoporosis. In view of this, early diagnosis and treatment are critical.

Currently, proteomics technology has identified how proteomics interact with osteoporosis. Proteomics technology research includes highly dynamic presentation modes of protein functional molecules, physiological and pathological phenomena. In serum samples of osteoporosis patients, Liang *et al*. analyzed the early stage of osteoporosis, identified protein expression differences for the first time^3^. A subsequent study reported that these differentially expressed proteins were mainly involved in biological processes, cell components and molecular functions, four proteins were candidates for early diagnosis of osteoporosis^4^. Furthermore, tandem mass tags (TMT) were used for liquid chromatography–tandem mass spectrometry (LC–MS) screening of body fluids, tissues and cells. To discover protein interaction biomarkers, String‐10.0 network analysis has long been considered of importance for diagnosis and treatment of postmenopausal osteoporosis^5^.

In fact, there are still some problems have not yet been addressed. It is well accepted that post‐translational modification (PTM) is a dynamic and reversible protein chemical modification that be involved in almost all processes of cells and plays an important regulatory role. Proteomic succinylation modification, a notable type of modification, is thought to impact metabolism, epigenetics and immunity. Zhang *et al*. (2011) first discovered lysine succinylation, a new type of post‐translational modification of proteins. Subsequently, western blotting analysis was used to identify the lysine succinylated peptides derived from the proteins in vivo^6^. Increasing evidence has revealed that succinylation locating on critical metabolic networks, such as enzyme activity, central metabolism pathway and cell stress response to aging and apoptosis^7^. As a result of the mitochondrial sirtuin SIRT5‐mediated lysine desuccinylation, lysine succinylation sites overlapped to differential extents and regulations compared with other diverse post‐translational modifications. Proteins potentially associated with biochemical activity and cellular respiration^8^. Bioinformatics have demonstrated that lysine succinylome, which participate in the regulation of multiple metabolic signaling pathways through the desuccinylase SIRT5, widely presented in mitochondrial energy metabolism regulators, as a global regulator^9^. Systematic profiling of metabolic interaction mainly concentrated on carbon metabolism, fatty acid metabolism and tricarboxylic acid (TCA) cycle, this viewpoint indicated signs of progress regarding treatment of osteoporosis^10^.

Proteome analysis have widely applied to the macro level of cells, tissues or organisms in the presence of all proteins and their modes of activity^11^. In that case, it appears significantly to be aware of the proteomic characteristics of osteoporosis. Medicine has already thrown up some positive effects in regard to improving BMD and reducing bone turnover markers mostly extracted from serum^12^. Based on these preceding studies, the purpose of the study focuses on succinyl‐proteome profiling in postmenopausal osteoporosis and osteopenia, providing potential targets for the treatment of osteoporosis.

## Materials and Methods

### *Materials*

Trypsin was purchased from Promega (Madison, USA). Acetonitrile was from Fisher Chemical (Waltham, USA). Trifluoroacetic acid, iodoacetamide, dithiothreitol, urea, and formic acid were from Sigma‐Aldrich (St. Louis, USA). H_2_O was from Thermo Fisher Scientific (Waltham, USA). A BCA kit was purchased from Beyotime Biotechnology (Shanghai, China).

### *Inclusion Criteria*

According to the diagnostic criteria recommended by the World Health Organization (WHO), the BMD of the lumbar spine was measured by dual‐energy X‐ray absorptiometry (Medlink, France): (i) BMD (T score ≥ −1 SD) was considered normal; (ii) BMD (−1 < T score ≤ −2.5 SD) as osteopenia; and (iii) BMD (T score < −2.5 SD) as osteoporosis. A total of 30 postmenopausal women aged from 55 to 70 years old were divided into three groups: Osteoporosis group, 10 cases; Osteopenia group, 10 cases; Normal group, 10 cases. All samples were screened from January 2016 to June 2018 (approved by the ethical committee of The Second Affiliated Hospital of Zhejiang Chinese Medical University on 9th March 2015).

### *Exclusion Criteria*

The exclusion criteria were as follows: (i) patients who did not meet the diagnostic criteria of osteoporosis (diabetes mellitus, Cushing's syndrome, thyroid or parathyroid function changes, osteomalacia, rheumatoid arthritis, multiple myeloma, bone tumors, osteoarthropathy, Paget's disease, osteogenesis imperfection, and other serious impacts on bone or calcium metabolism disease); (ii) those who had taken estrogen, steroid hormones, calcitonin, parathyroid hormones, diphosphate, fluoride, vitamin D, anticonvulsants, diuretics, and other drugs that affect bone metabolism) in the latest 6 months; (iii) those with cardiovascular, cerebrovascular, and hematopoietic system and other serious primary diseases; (iv) those with severe liver and kidney dysfunction, and mental illness patients.

### *Study Design*

According to screening proteomics from selected cases, without drug intervention, blood samples were taken from 30 cases once. Three comparison groups were designed to identify the succinylation sites and differentially expressed proteins, and to discover target proteins related to the diagnosis of osteoporosis and osteopenia.

### *Protein Extraction*

Serum was collected in the morning, centrifuged at 4°C for 10 min in 3000 *g*/min, then washed three times on ice using a high intensity ultrasonic processor (Scientz) in lysis buffer (8 M urea, 1% protease inhibitor cocktail). The remaining debris was removed by centrifugation at 12 000 *g* at 4°C for 10 min. As Osteoporosis/Normal, Osteopenia/Normal, and Osteoporosis/Osteopenia comparison groups, the supernatant was collected and the protein concentration was determined with the BCA kit according to the manufacturer's instructions.

### *Trypsin Digestion*

We added 8 M urea to the samples and 5 mM dithiothreitol to reduce the solution at 56°C for 30 min; then the samples were alkylated with 11 mM iodoacetamide for 15 min at room temperature in darkness. Finally, the samples were diluted by NH_4_HCO_3_ to ensure that the urea concentration was less than 2 M. For the first digestion, trypsin was added at 1:50 trypsin‐to‐protein mass ratio overnight, and for the second digestion at 1:100 trypsin‐to‐protein mass ratio for 4 h^13^.

### *High Performance Liquid Chromatography Fractionation*

We prepared high pH reverse‐phase high performance liquid chromatography for tryptic peptides fraction as previously described, using Thermo Betasil C18 column (5‐μm particles, 10‐mm ID, 250‐mm length). The operation was as follows: the gradient of peptides fraction was acetonitrile (8%–32%, pH = 9), 60 components were separated over 60 min, and then the peptides were merged into three components dried by vacuum centrifuging for subsequent operations.

### *Affinity Enrichment*

The peptides were dissolved in IP buffer solution (100 mM NaCl, 1 mM EDTA, 50 mM Tris–HCl, 0.5% NP‐40, pH 8.0), with the pre‐washed succinylated resin (product number: PTM‐402, from PTM Bio, China); the clear supernatant was incubated at 4°C overnight and then gently shaken. The IP buffer solution was reused at least four times and H_2_O was reused twice; then the bound peptides were eluted from the beads with 0.1% trifluoroacetic acid three times in total. Eventually, the elution was collected and vacuum‐dried^14^. In accordance with desalting steps according to C18 ZipTip instructions, perioperative management was essential for the liquid‐mass spectrometry analysis.

### *Liquid Chromatograph–Mass Spectrometer/Mass Spectrometer Analysis*

Tryptic peptides consisted of solvent A (dissolved by 0.1% formic acid) and solvent B (dissolved by 0.1% formic acid in 98%). LC–MS/MS analysis was performed with the EASY‐nLC 1000 Ultra Performance Liquid Chromatography (UPLC) system. Liquid phase gradient setting: 0–38 min, 6%–26% B; 38–52 min, 26%–36% B; 52–57 min, 36%–80% B; 57–60 min, 80% B, current speed maintained at 350 nL/min.

The peptides were separated by an ultra‐high‐performance liquid phase system, subjected by Nano‐spray ionization (NSI) ion source and then analyzed by Orbitrap Fusion (Thermo, China) mass spectrometry^15^. The ion source electrospray voltage applied was 2.0 kV. The peptide precursors and their secondary fragments were detected and analyzed in Orbitrap. For samples with scans, the primary MS scan range was 350–1550m/z and the resolution was 60 000; fixed primary mass was 100 m/z. Data‐dependent scanning (DDA) was used in this program. To improve the effective utilization of the MS, we set the automatic gain control threshold as 1.5E4, the signal threshold as 5000 ions/s, the maximum injection time as 70 MS, and the dynamic exclusion time as 30.0 s.

### *Database Search*

For convenience of reference, incorporated MS/MS data were progressed using Maxquant (v1.5.2.8, http://www.maxquant.org/)^16^. The database in the present study was Swiss‐Prot human (20 203 sequences), in which Trypsin/P was specified as the cleavage enzyme, allowing up to four missing cleavages. The minimum length of the peptide contained seven amino acid residues and the maximum number of peptides was five. The range of error tolerance varied in the first search (20 ppm) and the main search (5 ppm). Respectively, the secondary fragment ion was 0.02 Da. The FDR for protein identification and PSM identification were adjusted to 1%.

### *Bioinformatics*

Gene ontology (GO) annotation proteome was derived from the UniProt‐GOA database (v.5.14–53.0, http://www.ebi.ac.uk/GOA/). At the beginning of annotation, the system converted the protein ID to UniProt ID, then matched the GO ID with UniProt ID, and retrieved the corresponding information from the UniProt‐GOA database. If there was no protein information in the UniProt‐GOA database, based on protein sequences, the InterProScan soft would be used to predict the GO function. Shortly afterwards, the proteins were classified by Biological Process, Cellular Component and Molecular Function^17^.

In terms of the multiple differentially expressed protein classification, cluster analysis offered the chance to authenticate the accuracy of potential connections and differences in specific functions^7^, we collected the functional classification information and obtained *P*‐values of the protein groups, we screened the functional classification for significant enrichment (*P*‐value <0.05) in at least one cluster. Immediately after, the screened data matrix was transformed by the function *x* = −log10 (*P* value), and then classified by *Z*‐transformation. The *Z*‐scores were analyzed by one‐way hierarchical clustering. Figures from the “gplots” R‐package “heatmap.2” function clearly demonstrated the cluster memberships.

**Figure 1:**
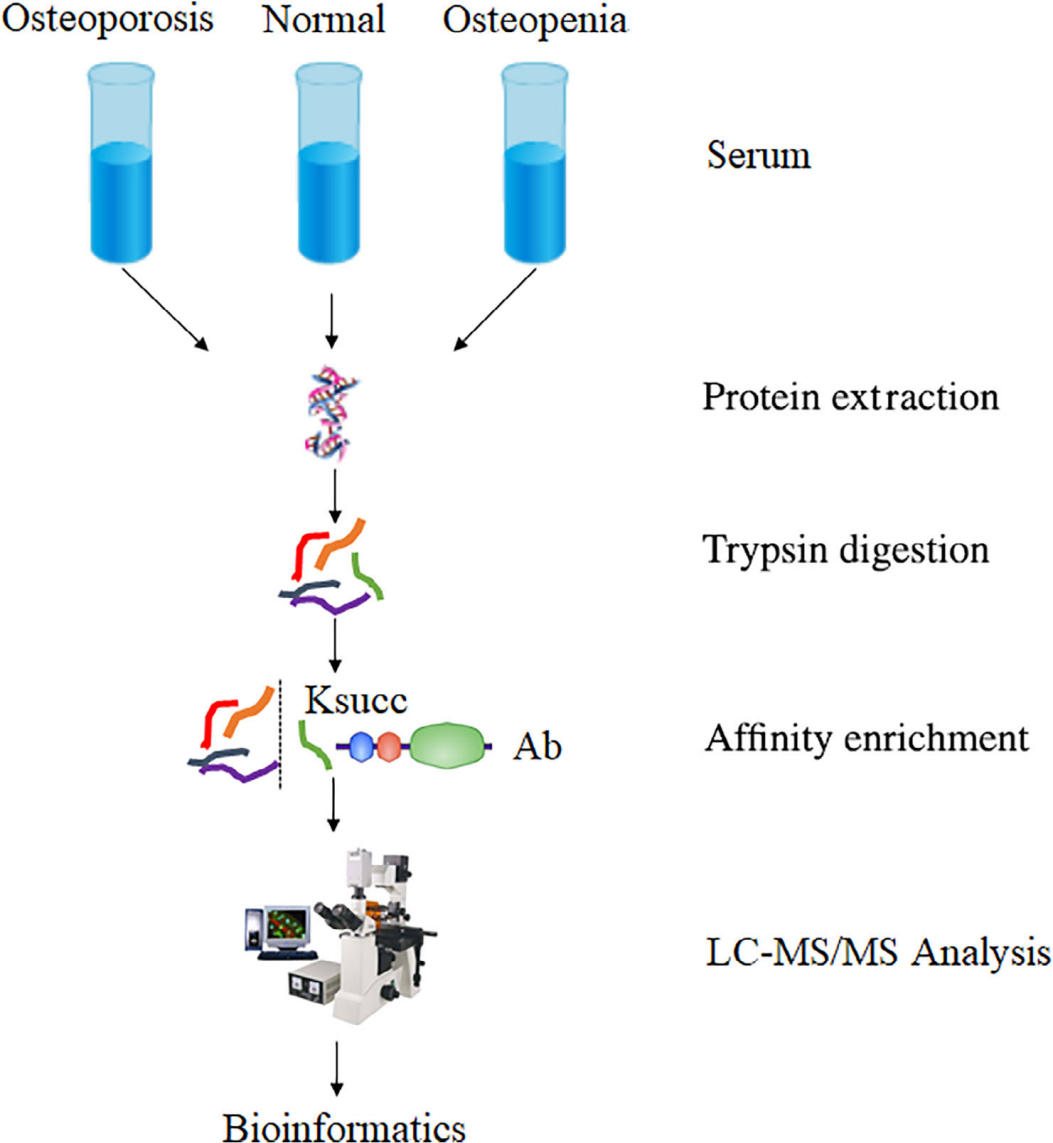
Overview of research process.

An overview of the research process is presented in Fig. 1.

## Results

**Figure 2:**
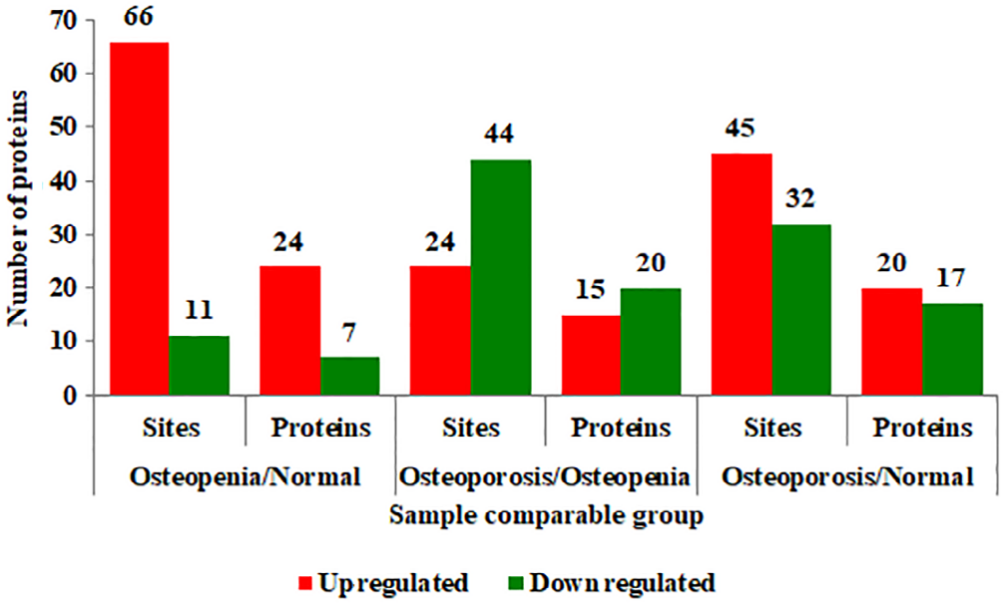
Numbers of quantified succinylation sites and proteins in the three comparison groups.

The average age of 30 cases was 63.28 ± 5.78 years, with no significant difference between groups (*P* > 0.05). A total of 113 succinylation sites on 35 proteins had quantitative information. The variation of the different multiple folds were more than 1.2 times as a significant increase for up‐regulated and less than 1/1.2 times as a significant decrease for down‐regulated. Among the quantified succinylation sites, 45 were up‐regulated and 32 down‐regulated in the Osteoporosis/Normal comparison group, 66 were up‐regulated and 11 down‐regulated in the Osteopenia/Normal comparison group, 24 were up‐regulated and 44 down‐regulated in the Osteoporosis/Osteopenia comparison group. Among the quantified succinylation proteins, 20 were up‐regulated and 17 down‐regulated in the Osteoporosis/Normal comparison group, 24 were up‐regulated and 7 down‐regulated in the Osteopenia/Normal comparison group, 15 were up‐regulated and 20 down‐regulated in the Osteoporosis/ Osteopenia comparison group (Fig. 2 **)**.

### *Subcellular Localization*

To characterize these differentially changed proteins, we used Wolfpsort software to predict and classify the subcellular localization (as shown in a localization map of the subcellular structure of up‐regulated and down‐regulated proteins). Table 1 illustrates that the proteins identified were most distributed in the following locations: extracellular, nucleus, mitochondria, endoplasmic reticulum, cytoplasm and cyto_nucl. The percentage of proteins differed in extracellular, nucleus and endoplasmic reticulum between up‐regulated and down‐regulated proteins.

**Table 1:** Subcellular structure of up‐regulated proteins and down‐regulated proteins

Comparison group	Subcellular location (number of proteins)						
	Extracellular	Endoplasmic reticulum	Plasma membrane	Nucleus	Cyto_nucl	Mitochondria	Cytoplasm
Osteopenia/Normal	15	1	0	7	0	2	3
Osteoporosis/Osteopenia	15	1	1	6	1	2	3
Osteoporosis/Normal	16	1	0	6	1	2	2

### *Functional Enrichment Analysis*

**Figure 3:**
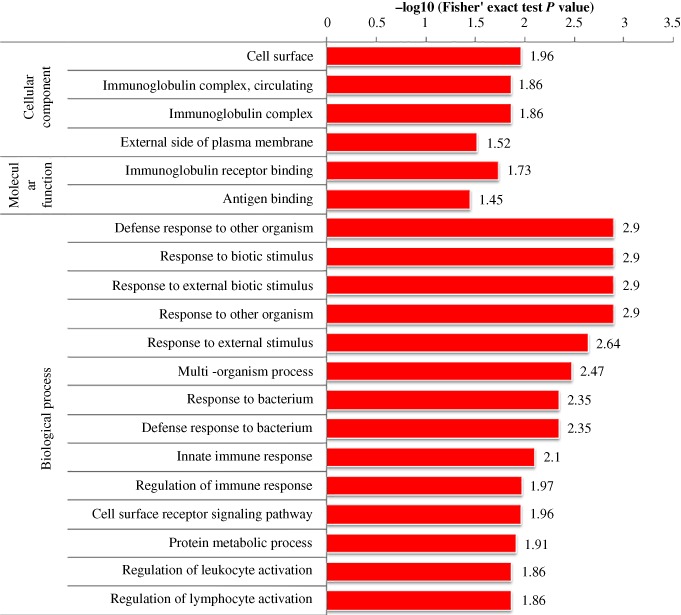
Functional enrichment analysis in Osteoporosis/Osteopenia comparison group.

**Figure 4:**
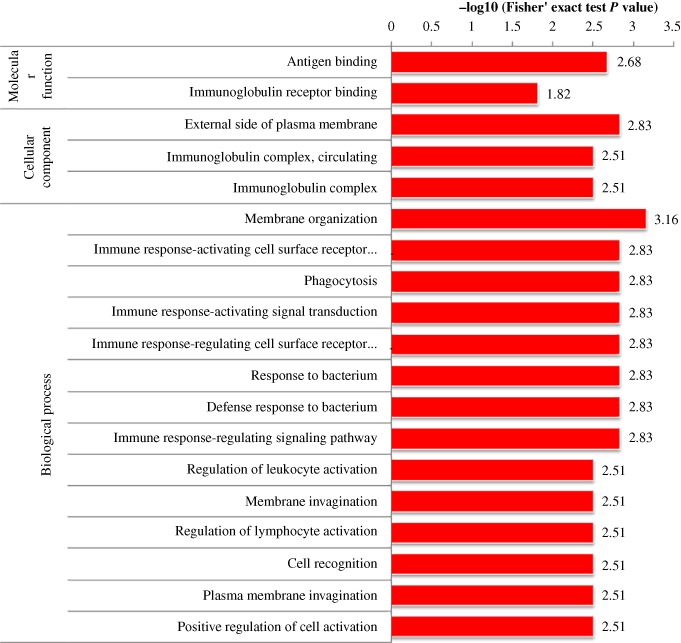
Functional enrichment analysis in Osteopenia/Normal comparison group.

According to the annotations for all identified proteins containing modified sites and corresponding proteins with differentially modified sites, we analyzed the GO domain to detect whether there was a significant enrichment trend of differential expression in some functional types. For enrichment tests, the *P*‐value obtained by Fisher's exact test was transformed into a negative logarithm (−log10), the *P*‐value obtained by Fisher's exact test was obtained. The larger the converted value, the more significant the enrichment of this function type would be Fig. 3, 4, 5 show that in multiple comparisons, the up‐regulated and down‐regulated proteins were highly enriched in the Biological Process response to organism transport and localization, endocytosis, signaling pathway, stimulus, biological regulation and immune system process; in Cellular Component, enriched in the process of cell surface, immunoglobulin complex and external region; in Molecular Function, enriched in the process of receptor binding, peptidase and endopeptidase activity.

**Figure 5:**
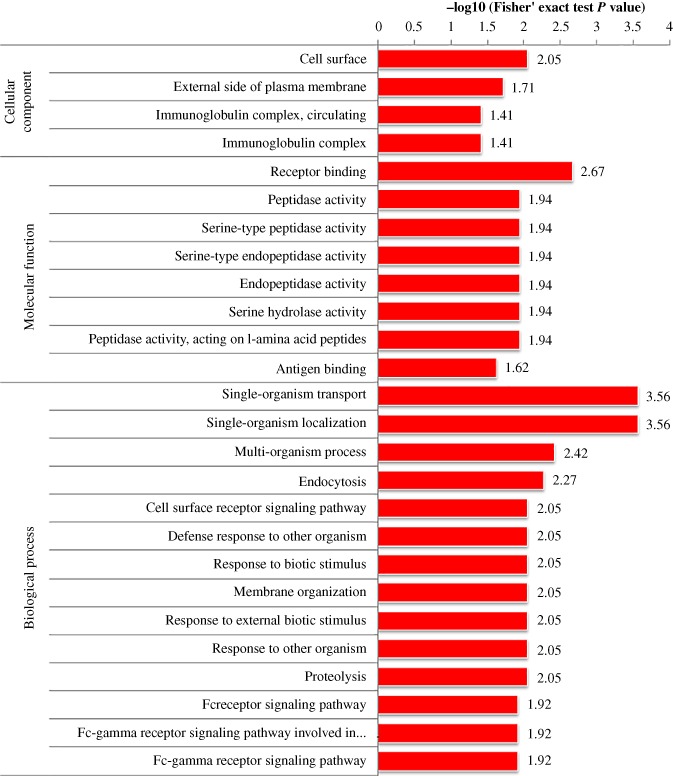
Functional enrichment analysis in Osteoporosis/Normal comparison group.

### *KEGG Pathway Enrichment*

**Figure 6:**
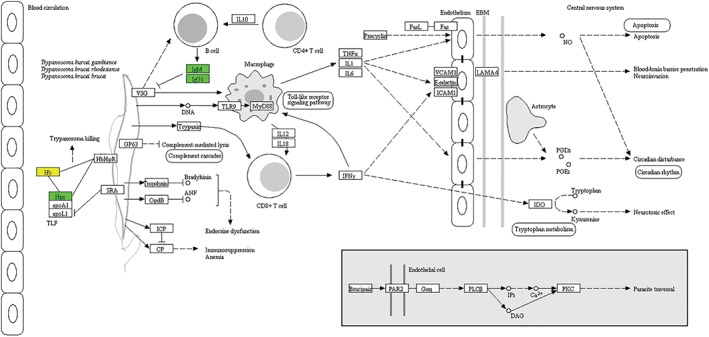
KEGG pathway enrichment in Osteopenia/Normal comparison group.

We visualized KEGG pathways from different comparison groups with significant enrichment of the corresponding proteins at differentially modified proteins. In Fig. 6 (Figure sourced from KEGG databases, and contributed by Kanehisa Laboratories), the green element indicates the down‐regulated level of modification proteins, while the yellow element indicates that there are many proteins at this node, including up‐regulated and down‐regulated proteins. It was found that haptoglobin was up‐regulated and down‐regulated in the three groups. Apolipoprotein A‐I was only down‐regulated in the Osteopenia/Normal group, while up‐regulated in the Ostoeporosis/Osteopenia and Ostoeporosis/Normal groups. Apolipoprotein A‐II was only up‐regulated in the Osteopenia/Normal group, while down‐regulated in the Ostoeporosis/Osteopenia and Ostoeporosis/Normal groups. Hemoglobin subunit alpha was only up‐regulated and down‐regulated in the Osteopenia/Normal group (Table 2).

**Table 2:** Significant enrichment of the corresponding differentially modified proteins from different comparison groups

Comparison group	Protein accession	Ratio	Regulated type	Protein names	Gene names	Score
Osteopenia/Normal	P00738	0.78	Down	Haptoglobin	HPT	96.866
Osteopenia/Normal	P00738	2.77	Up	Haptoglobin	HPT	108.14
Osteopenia/Normal	P00738	2.1	Up	Haptoglobin	HPT	129.42
Osteopenia/Normal	P00738	1.24	Up	Haptoglobin	HPT	120.63
Osteopenia/Normal	P00738	0.63	Down	Haptoglobin	HPT	126.71
Ostoeporosis/Osteopenia	P00738	2.52	Up	Haptoglobin	HPT	96.866
Ostoeporosis/Osteopenia	P00738	0.69	Down	Haptoglobin	HPT	120.63
Ostoeporosis/Normal	P00738	1.57	Up	Haptoglobin	HPT	96.866
Ostoeporosis/Normal	P00738	0.77	Down	Haptoglobin	HPT	126.71
Osteopenia/Normal	P02647	0.76	Down	Apolipoprotein A‐I	APOA1	99.343
Osteopenia/Normal	P02647	0.66	Down	Apolipoprotein A‐I	APOA1	125.57
Osteopenia/Normal	P02647	0.42	Down	Apolipoprotein A‐I	APOA1	86.367
Ostoeporosis/Osteopenia	P02647	1.24	Up	Apolipoprotein A‐I	APOA1	99.343
Ostoeporosis/Normal	P02647	1.41	Up	Apolipoprotein A‐I	APOA1	99.343
Ostoeporosis/Normal	P02647	1.94	Up	Apolipoprotein A‐I	APOA1	125.57
Ostoeporosis/Normal	P02647	2.45	Up	Apolipoprotein A‐I	APOA1	86.367
Ostoeporosis/Normal	P02647	1.54	Up	Apolipoprotein A‐I	APOA1	65.809
Osteopenia/Normal	P02652	1.79	Up	Apolipoprotein A‐II	APOA2	115.91
Ostoeporosis/Osteopenia	P02652	0.37	Down	Apolipoprotein A‐II	APOA2	115.91
Ostoeporosis/Normal	P02652	0.62	Down	Apolipoprotein A‐II	APOA2	115.91
Ostoeporosis/Normal	P02652	2.69	Up	Apolipoprotein A‐II	APOA2	111.06
Osteopenia/Normal	P69905	2.03	Up	Hemoglobin subunit alpha	HBA	77.899
Osteopenia/Normal	P69905	0.5	Down	Hemoglobin subunit alpha	HBA	100.45

### *Cluster Analysis*

**Figure 7:**
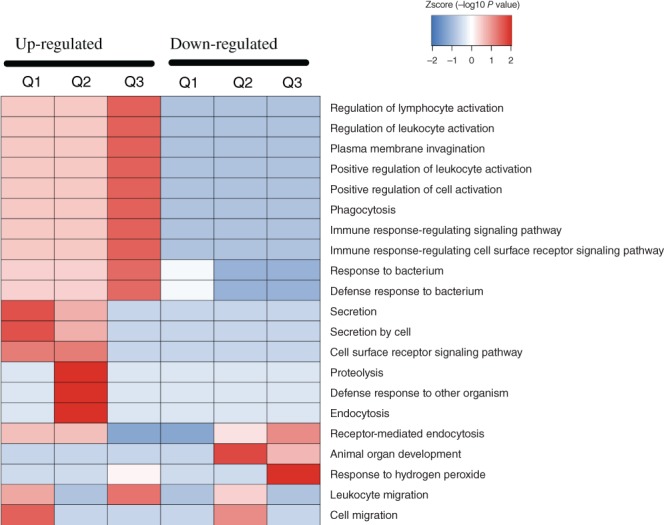
Biological process.

**Figure 8:**
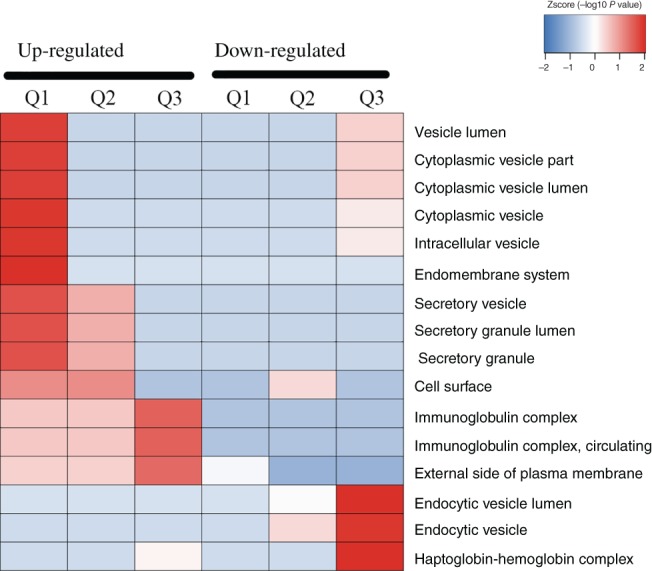
Cellular component.

**Figure 9:**
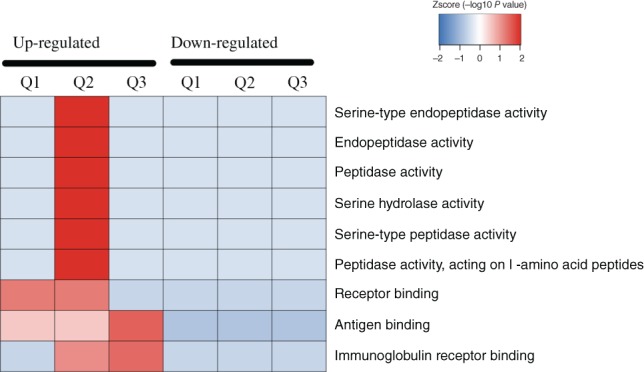
Molecular function.

Based on GO enrichment, the cluster analysis thermal map includes Biological Process (A), Cellular Component (B), and Molecular Function (C). Quantitative proteins of three comparison groups (Q1 as Osteoporosis/Osteopenia, Q2 as Osteoporosis/Normal, Q3 as Osteopenia/Normal) cluster analysis were performed. The Biological Process (Fig. 7) demonstrated that the up‐regulated Q1–3 proteins were highly enriched in response to immune response, signaling pathway, proteolysis, lymphocyte, leukocyte and cell activation, which might relate to the pathogenesis of osteoporosis. The Cellular Component (Fig. 8) indicated that the up‐regulated Q1–3 proteins were highly enriched as follows: immunoglobulin complex, circulating and external side of plasma membrane. The Molecular Function (Fig. 9) indicated that the up‐regulated Q1–3 proteins were highly enriched in receptor binding, antigen binding, and immunoglobulin receptor binding.

## Discussion

### *Succinyl‐proteome Characteristic*

Currently, compared with methylation and acetylation, lysine succinylation could induce more changes in protein properties, structure and function^18^. In the oxidative phosphorylation pathway, succinylation had been preferred as the SIRT5 target^19^. Lysine succinylation might regulate the pentose phosphate pathway and the endoplasmic reticulum protein processing pathway in their core enzymes^20^. Moreover, lysine succinylation in *Pseudomonas aeruginosa* gave some targets to develop effective antibacterial agents^21^. Evidence showed that succinylation was also involved in the establishment of osteoporosis, and a vitamin D receptor knockout animal model was used to demonstrate this possibility, these experimental results not only indicated that 209 sites of 159 proteins were up‐regulated and 3 sites of 3 proteins were down‐regulated but also confirmed eight distinct motifs around the succinylation site^10^.

### *Modification Field*

Results showed that the effective proteins covered the endocrine system, signal transduction, the sensory system, transcription, signal molecules and interaction, significantly enriched membrane transportion. Meanwhile, more approaches are needed to provide robust evidence. Tannahill *et al*. analyzed the succinate enhanced interleukin‐1 beta production during inflammation^22^. Besides, whether succinate led to net inflammation or anti‐inflammation depending on the cellular context^23^, the research would have been more relevant if a wider range of metabolic disease models had been explored.

### *Osteopenia*

Osteopenia, a preclinical state of osteoporosis, is characterized by slight loss of bone mass with an annual rate of 0.4%–0.6%. Approximately, this rate accelerates to 1.5%–2.5% in postmenopausal women^24^. Imbalance between bone resorption and bone formation, abnormity of bone metabolism markers, specification of signaling pathway, and biological information are involved in this stage. Prevention and treatment of osteopenia are significant in reducing the incidence of osteoporosis. Further protein modification (e.g. disrupted histone acetylation) in BMSC leads to bone formation defects during osteoporosis^25^. Pathways directly related to bone metabolism, such as osteoclast differentiation and calcium absorption, might help to determine the proteins composition in osteoporosis. In this study, we noticed that an imbalance in lipid metabolism might alter bone mass and quality. Hemoglobin subunit alpha was screened for differentially expressed proteins. Hemoglobin, as known for a special protein transports oxygen in red blood cells, its subunit proteasome degradation might take oxidatively damaged to hemoglobin, especially for hematopoietic tissue and non‐chondrocyte^26^. Different hemoglobin subunits express oxygen affinity through peroxiredoxin 2, and alpha‐subunit‐specific modifying agents could increase oxygen binding^27^, ^28^. Succinylation was involved in the regulation of enzymatic activity, central metabolism, cellular response to aging and apoptosis in vivo^7^. Through enrichment in succinylation modification, hemoglobin subunit alpha was screened in osteopenia.

### *Key Factors and Corresponding Pathways*

Interestingly, in the Osteopenia/Normal comparison group, apolipoprotein A‐I was down‐regulated and apolipoprotein A‐II was up‐regulated, but the concentrations bucked the trend in the other two groups. Apolipoprotein A‐I deficiency increased adipocytes and reduced the production of osteoblasts, resulting in bone loss, bone mass loss^29^, and bone metastasis microenvironment^30^. According to Chaput *et al*.^31^, apolipoprotein A‐I was the most significant variation of proteins in patients with osteopenia and osteoarthritis. In addition, apolipoprotein A‐II was thought to be correlated to lipid and glucose metabolism^32^. Apolipoprotein A‐II decreased by a mean of 24% with anabolic steroid therapy for postmenopausal osteoporosis and reduced high density lipoproteins^33^. Perhaps the lower level of apolipoprotein A‐II significantly contributed to bone mass and bone metabolic indices, but untill now it remains uncertain. According to animal experiments and clinical data, apolipoproteins A‐I and A‐II blocked IL‐1 or tumor necrosis factor (TNF) ligand/counter‐ligand interaction related to bone resorption^34^. Furthermore, the apolipoprotein A‐II/A‐I ratio could be a useful biochemical marker in rheumatoid arthritis^35^.

For the records, haptoglobin was identified in blood samples as a risk factor for postmenopausal osteoporosis^36^. These screened proteins played a particular role in the toll‐like receptor (TLR) signaling pathway. TLR, a remarkable protein and mediated activation of innate immunity, hosted not only defense against pathogens but also immune disorders. Anti‐inflammatory activity *via* suppression of nuclear factor‐kappa B pathway and mitogen‐activated protein kinase (MAPK) signaling pathway has been reported, the most noteworthy of the pathways lied in they were mediated by TLR^37^. In addition, the experssion of inflammatory factors, such as interleukin (IL)‐4, IL‐6 and interferon (IFN)γ varied with unique trends^38^. Here we found that TNFα, IL‐1, IL‐6 and IFNγ were related to this process, that might be the major reason for the likelihood of osteocytes apoptosis.

### *Apoptosis of Osteocytes*

Apoptosis activity, a process of autonomous death in cells, was considered to be closely related to hormones. Lackness of estrogen in postmenopausal women would give rise to irregular apoptosis activity, ultimately leading to osteoporosis. Estrogen depletion resulted in osteocyte apoptosis, which stimulated osteoclasts, caused bone resorption and loss^39^. Due to excess glucocorticoids induced osteoporosis, apoptosis of bone marrow‐derived mesenchymal stem cells (BMSC) had been corroborated precisely^40^. Anti‐apoptosis requires further research. A study was designed to verify alpinumisoflavone's inhibition against dexamethasone‐induced apoptosis in osteoblastic and osteocytic cells^41^,which better explained the abovementioned issue. There was another opinion that osteopontin inhibited apoptosis, accelerated T helper 1 immune response and cell survival^42^.

### *Study Limitations*

There were also some deficiencies in this study. The number of participant proteins and sites were relatively low, besides, the expression levels required further verification. Although we had made use of Motif analysis, yet obtained no data. Establishing a larger samples of clinical trials for further study might credibly elucidate this area of research.

### *Conclusion*

In this study, discovery and identification from serum for differential sites, proteins and potential efficacies on bone remodeling provided invaluable evidence that succinyl‐proteome was related to occurrence of postmenopausal osteoporosis. Multiple factors and pathways seemed to be involved in the process, ranging from bone formation to resorption. These results demonstrated from functional analysis promoted apolipoprotein A‐I, apolipoprotein A‐II, hemoglobin subunit alpha, and haptoglobin mediated with TLR, TNF α, IL‐4, IL‐6 and IFN γ during apoptosis mechanism, could be potential avenues for prevention, treatment and monitoring in osteopenia. In the following studies, we will continue to verify the expression of these proteins by western blotting and ELISA. In addition, a larger sample instead of a small would be required to recognize the relationship among other differentially expressed proteins in postmenopausal women with osteoporosis and osteopenia.
